# No ergogeniceffect of β-alanine on repeated sprint ability: a systematic review and multilevel meta-analysis of randomized controlled trials

**DOI:** 10.3389/fnut.2026.1818755

**Published:** 2026-03-26

**Authors:** Weibao Liang, Dandan Kong, Yiqiang Wang, Tianyuan Yu, Mingmei Chen, Wenbai Huang

**Affiliations:** 1 Guangdong Provincial Key Laboratory of Speed Capability Research, Su Bingtian Center for Speed Research and Training, School of Physical Education, Jinan University, Guangzhou, China; 2 School of Art Design and Media, Guangzhou Xinhua University, Guangzhou, China; 3 School of Sports Training, Xi’an Physical Education University, Xi’an, China; 4 School of Information Technology, Vitebsk State University named after P.M. Masherov, Vitebsk, Republic of Belarus; 5 School of Economics and Finance, Zhanjiang University of Science and Technology, Zhanjiang, China

**Keywords:** beta-alanine, carnosine, ergogenic aids, high-intensity interval training, multilevel meta-analysis, repeated sprint ability

## Abstract

**Objective:**

Chronic beta-alanine supplementation is a prevalent nutritional strategy to augment intracellular buffering capacity via elevated muscle carnosine. While its ergogenic efficacy in continuous, high-intensity exercise is established, its impact on repeated sprint ability (RSA)—governed by extremely brief work bouts and phosphocreatine (PCr) kinetics—remains equivocal.

**Methods:**

A systematic search of PubMed, Web of Science, Scopus, Cochrane Library, Embase and SPORTDiscus was conducted up to January 2026 (PROSPERO: CRD420261304011). Eligible studies included randomized controlled trials (RCTs) investigating oral beta-alanine supplementation (≥ 2 weeks) versus placebo on RSA in healthy populations. Outcomes were standardized into Standardized Mean Differences (SMD, Hedges’ g). To account for the statistical dependency of multiple effect sizes extracted from individual cohorts, a multilevel random-effects model was employed. Methodological quality was assessed using the Cochrane RoB 2.0 tool, and the certainty of evidence was evaluated via the GRADE framework.

**Results:**

Among 17 included RCTs, the meta-analysis revealed no statistically significant improvements in Mean RSA Performance (SMD = −0.018, 95% CI [−0.207, 0.170], *p* = 0.841), Peak RSA Performance (SMD = 0.205, 95% CI [−0.073, 0.483], *p* = 0.130), or Fatigue Decrement (SMD = −0.020, 95% CI [−0.516, 0.476], *p* = 0.929). Furthermore, subgroup and meta-regression analyses confirmed these null findings were not significantly moderated by total administered dose, duration, exercise modality, or training status.

**Conclusion:**

In conclusion, chronic β-alanine supplementation does not provide a clear improvement in total work capacity, maximal anaerobic power, or fatigue resistance during repeated sprinting. The augmented intracellular buffering from carnosine may be insufficiently aligned with the acute metabolic demands of RSA, which is primarily dictated by PCr depletion and oxidative recovery rather than maximal glycolytic acidosis.

**Systematic review registration:**

https://www.crd.york.ac.uk/PROSPERO/view/CRD420261304011, identifier CRD420261304011.

## Graphical Abstract

**Figure:**
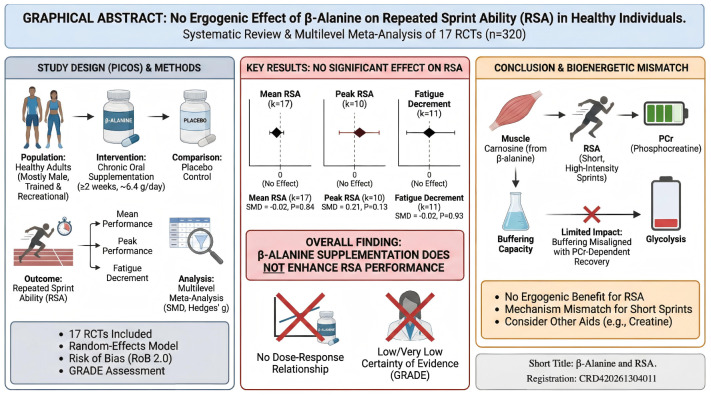
Study design (Left): Overview of the systematic review and multilevel meta-analysis (17 RCTs) evaluating the effects of chronic $\beta$-alanine supplementation versus placebo on Repeated Sprint Ability (RSA) in healthy adults. Key Results (Center): Forest plot summaries demonstrate no significant ergogenic effect of beta-alanine across three key RSA dimensions: mean performance, peak performance, and fatigue decrement (*p* > 0.05). No dose-response relationship was found. Conclusion and Bioenergetic Mismatch (Right): Illustrates the physiological mechanism behind the null findings. The enhanced buffering capacity from beta-alanine (muscle carnosine) does not align with the phosphocreatine (PCr)-dependent recovery demands of short, high-intensity repeated sprints, concluding no overall benefit for this specific exercise modality. Created in BioRender. Liang, W. (2026) https://BioRender.com/vad19s6.

## 1 Introduction

Repeated sprint ability (RSA) is a defining performance attribute in intermittent sports, characterized by repeated maximal or near-maximal efforts separated by brief recovery, with an ensuing decline in mechanical output across bouts (1, 2). The physiological basis of this decrement is inherently time-dependent and multifactorial: early sprints rely heavily on rapid ATP turnover supported by intramuscular phosphocreatine (PCr), whereas incomplete recovery progressively constrains PCr restoration and increases reliance on glycolysis as repetitions accumulate (3, 4). Importantly, mechanistic evidence indicates that performance loss during repeated maximal efforts reflects an integrated disturbance in muscle function—encompassing substrate availability, metabolite/ionic perturbations, excitation–contraction coupling, and only later, more pronounced acid–base disruption—rather than a single dominant metabolite (5–7).

Within this context, chronic β-alanine supplementation is widely used to increase intramuscular carnosine, thereby augmenting intracellular buffering capacity and potentially attenuating exercise-induced acid–base perturbation (8). Human studies demonstrate that several weeks of daily β-alanine supplementation reliably elevates muscle carnosine content, providing a plausible mechanistic basis for improving performance when high glycolytic flux and proton accumulation are salient constraints (8, 9). Consistent with this mechanism, contemporary meta-analytic evidence indicates the most reproducible ergogenic effects in high-intensity exercise tasks of approximately 1–4 min, where acid–base perturbation is more likely to be performance-limiting (10).

However, whether enhanced buffering meaningfully translates to RSA remains uncertain. A key biological consideration is that sustaining repeated-sprint performance depends strongly on inter-bout recovery processes, particularly the kinetics of PCr resynthesis. Biopsy-based repeated-sprint experiments show that recovery of subsequent sprint performance is tightly associated with PCr resynthesis even when muscle pH remains depressed, suggesting that acid–base normalization is not a prerequisite for short-term restoration of repeated-sprint capacity (11, 12). This raises the possibility that β-alanine–mediated buffering, while mechanistically coherent for longer glycolytically dominated tasks, may exert limited influence on the dominant recovery constraints governing many RSA formats.

Interpretation of the existing trial literature is additionally complicated by methodological heterogeneity in RSA protocol design and, critically, in how “fatigue” is quantified. Reliability/validity work demonstrates that commonly used fatigue indices can vary substantially in measurement properties and inference, even when derived from the same sprint series (13). Moreover, RSA trials frequently report multiple correlated outcomes from the same participants (e.g., peak power, mean power, and fatigue metrics), and treating these effect sizes as independent can underestimate standard errors and overstate precision. Robust evidence-synthesis methods—such as robust variance estimation—are specifically designed to address dependent effect sizes in meta-regression and improve inferential validity (14).

Therefore, this systematic review used a multilevel meta-analytic approach to quantify the effect of chronic β-alanine supplementation on RSA while explicitly addressing non-independence arising from multiple, correlated outcomes reported within the same trials—an issue that can otherwise artificially narrow uncertainty and inflate precision (14). We synthesized outcomes by functional domain (mean performance, peak performance, fatigue decrement) and tested prespecified moderators (total dose, supplementation duration, training status) to clarify when, if ever, β-alanine meaningfully improves RSA.

## 2 Materials and methods

### 2.1 Protocol and registration

This systematic review and multilevel meta-analysis was conducted in strict adherence to the Preferred Reporting Items for Systematic Reviews and Meta-Analyses (PRISMA) 2020 guidelines. The study protocol was prospectively registered in the International Prospective Register of Systematic Reviews (PROSPERO) under the registration number CRD420261304011.

### 2.2 Literature search strategy

A comprehensive and systematic literature search was performed across six electronic databases: PubMed, Web of Science, Scopus, Cochrane Library, Embase and SPORTDiscus, from their inception to January 2026 (Supplementary Table S1). The Boolean search strategy utilized a combination of Medical Subject Headings (MeSH) and free-text terms related to the intervention and outcomes. The primary search string included: (“beta-alanine” OR “beta-alanine” OR “carnosine”) AND (“repeated sprint*” OR “RSA” OR “intermittent sprint*” OR “high-intensity interval* “OR “multiple sprint*”). To ensure literature saturation, the reference lists of all included articles and relevant narrative reviews were manually screened for additional eligible trials.

### 2.3 Eligibility criteria

The inclusion and exclusion criteria were established based on the PICOS (Population, Intervention, Comparison, Outcomes, and Study Design) framework:

Population (P): Healthy individuals, encompassing recreationally active subjects and highly trained/elite athletes. Studies involving elderly populations, animals, or individuals with clinical pathologies were excluded.

Intervention (I): Chronic oral beta-alanine supplementation with a loading phase of at least 2 weeks. Trials employing acute single-dose supplementation or co-supplementation strategies (e.g., beta-alanine combined with creatine or sodium bicarbonate) were excluded unless a distinct, isolated beta-alanine treatment arm was available.

Comparison (C): A matched placebo group (e.g., dextrose, maltodextrin, or cellulose).

Outcomes (O): The study must report at least one quantitative measure of Repeated Sprint Ability (RSA). To account for methodological heterogeneity across different exercise modalities (e.g., running, cycling, swimming), outcomes were harmonized into three functional dimensions: Mean RSA Performance (e.g., total sprint time, mean power output), Peak RSA Performance (e.g., best sprint time, peak power), and Fatigue Decrement (e.g., fatigue index, percentage decrement score).

Study Design (S): Randomized Controlled Trials (RCTs) utilizing either parallel or crossover designs.

### 2.4 Data extraction

Two independent reviewers screened the titles, abstracts, and full texts of the retrieved articles. Any discrepancies were resolved through discussion or consultation with a third reviewer. Extracted data included: study characteristics (authors, year, sample size, sex, age, training status), supplementation protocols (daily dose, duration, total dose, formulation type), RSA protocol details (exercise mode, number of sets/repetitions, rest intervals), and specific outcome measures (pre- and post-intervention means, standard deviations, or changes from baseline). When data were only presented graphically, WebPlotDigitizer (version 4.6) was used for precise extraction. Corresponding authors were contacted when necessary data were missing.

### 2.5 Risk of bias and certainty of evidence assessment

The methodological quality and risk of bias for each included RCT were evaluated by two independent reviewers using the Cochrane Risk of Bias 2.0 (RoB 2) tool. Studies were assessed across five domains: randomization process, deviations from intended interventions, missing outcome data, measurement of the outcome, and selection of the reported result. The overall certainty of the evidence for each functional RSA outcome was subsequently appraised using the Grading of Recommendations Assessment, Development and Evaluation (GRADE) framework, which considers risk of bias, inconsistency, indirectness, imprecision, and publication bias.

### 2.6 Statistical analysis

Given the diverse measurement units (e.g., seconds, watts, percentages), effect sizes were calculated as Standardized Mean Differences (SMD, specifically Hedges’ g to correct for small-sample bias) with 95% Confidence Intervals (CIs). For temporal variables where lower values indicate better performance (e.g., total sprint time), the means were mathematically inverted (multiplied by −1) prior to analysis to ensure a consistent direction of effect across all forest plots.

To appropriately address the statistical dependency arising from extracting multiple effect sizes from a single study (e.g., multiple time points, distinct subgroups, or multiple measures of the same construct), a multilevel random-effects meta-analysis model was employed. Effect sizes were nested within their respective primary studies, thereby robustly partitioning the variance and mitigating artificially deflated standard errors. Between-study heterogeneity was quantified using the I^2^ statistic.

Potential sources of variance were explored through categorical subgroup analyses (duration ≤ 4 vs. > 4 weeks; exercise modality; training status) and continuous random-effects meta-regression (total administered dose in grams). The robustness of the pooled estimates was evaluated via a leave-one-out sensitivity analysis. Finally, potential publication bias and small-study effects were assessed visually using funnel plots and quantitatively using Egger’s regression test, Begg’s rank correlation test, and the Duval and Tweedie’s trim-and-fill method. All statistical analyses were performed using R software (R 4.5.2), with a pre-determined alpha level of *P* < 0.05 defining statistical significance. Data visualization (including Orchard plots) was generated using the orchaRd and robvis packages.

## 3 Results

### 3.1 Study selection and characteristics

**FIGURE 1:**
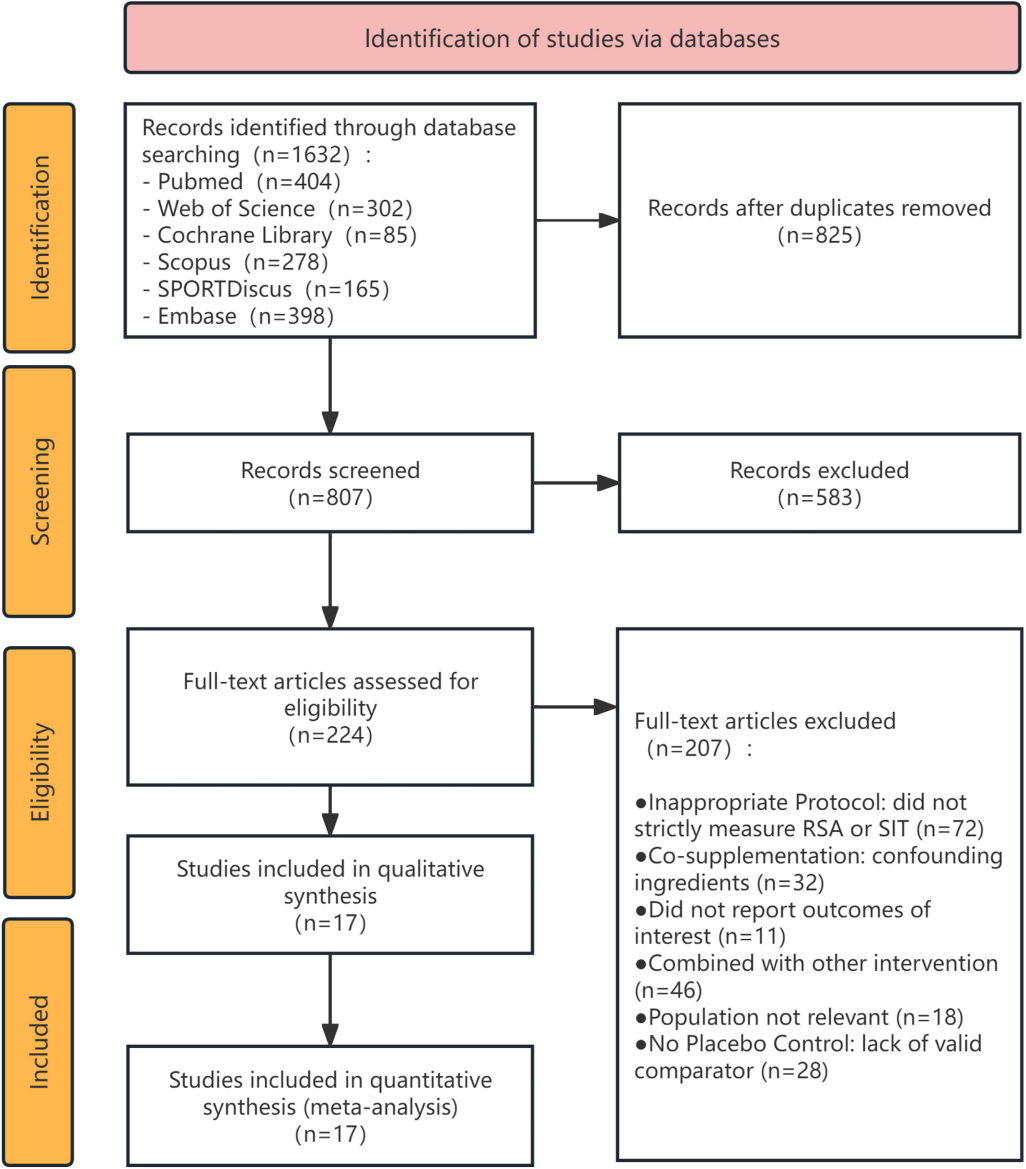
PRISMA flow diagram of the study selection process. The flowchart details the identification, screening, eligibility, and inclusion phases for the systematic review and multilevel meta-analysis of randomized controlled trials investigating β-alanine supplementation and repeated sprint ability.

The systematic literature search and selection process yielded 17 randomized controlled trials (15–31) that fulfilled the inclusion criteria for qualitative and quantitative synthesis (Figure 1). The characteristics of the included studies and their respective Repeated Sprint Ability (RSA) protocols are summarized in Table 1. The total aggregated sample comprised primarily young adults (mean age ranging from 16 to 28 years). The majority of the studies recruited male participants, while three studies (21, 24, 25) exclusively involved females. Participants exhibited diverse training backgrounds, ranging from recreationally active individuals to elite athletes (e.g., combat soldiers, U20 elite footballers, and trained cyclists). The exercise modalities utilized for RSA testing varied appropriately according to the participants’ athletic disciplines, including running (*k* = 9), cycling (*k* = 6), and swimming (*k* = 2). The RSA protocols demonstrated substantial methodological diversity, with sprint repetitions ranging from 4 to 12 per set, and rest intervals spanning from a highly demanding 10 s to 4 min.

**TABLE 1:** Characteristics of the included studies and repeated sprint ability (RSA) protocols.

Study	Sample size (BA/PLA)	Sex	Age (y)	Training status	Exercise mode	RSA protocol (Sets × Reps × Dist/Time)	Rest interval	Key outcomes
Brisola et al. (15)	11/11	M	18 ± 2	Water Polo Players	Swimming	2 × (6 × 10-m)	17 s	Total Time
Claus et al. (16)	8/7	M	16 ± 2	Junior Water Polo	Swimming	1 × (8 × 15-m)	30 s	Total Time, Mean Velocity
Cochran et al. (17)	12/12	M	23 ± 2	Recreationally Active	Cycling	1 × (4–6 × 30 s)	4 min	Mean Power, Total Work
Danaher et al. (18)	8 (Cross)	M	24	Recreationally Active	Cycling	1 × (5 × 6 s)	24 s	Total Work, Peak Power
Ducker et al. (19)	6/6	M	24	Team Sport Athletes	Running	3 × (6 × 20-m)	25 s	Total Sprint Time
Hoffman et al. (20)	9/9	M	19.9 ± 0.8	Combat Soldiers	Running	1 × (5 × 30-m)	30 s	Average Sprint Time
Kresta et al. (21)	8/7	F	22 ± 3	Recreationally Active	Cycling	1 × (4 × 30 s)	2 min	Mean Power, Peak Power
Milioni et al. (22)	13/14	M	17 ± 1	Basketball (U17)	Running	1 × (10 × 30-m)	30 s	Total Time, Fatigue Index
Milioni et al. (23)	9/9	M	26 ± 5	Physically Active	Running	2 × (6 × 35-m)	10 s	Mean Power, Total Time
Ribeiro et al. (24)	12/12	F	18 ± 1	Elite Footballers (U20)	Running	1 × (6 × 35-m)	10 s	Mean Power, Total Time
Rosas et al. (25)	8/8	F	23.7 ± 2.4	Amateur Soccer	Running	1 × (6 × 40-m)	20 s	Mean Time, Fatigue Index
Saunders et al. (26)	18/18	M	22 ± 3	Team Sport Athletes	Running	LIST (Variable)	Active	Mean 15-m Sprint Time
Saunders et al. (27)	8/8	M	25 ± 3	Recreationally Active	Running	3 × (5 × 6 s)	24 s	Mean Power, Peak Power
Smith et al. (28)	7/8	M	21.0 ± 1.8	Rugby Players	Running	1 × (6 × 30 s)	30 s	Total Distance
Sweeney et al. (29)	9/10	M	21 ± 2	Physically Active	Running	2 × (5 × 5 s)	45 s	Mean Power, Fatigue Index
Wang et al. (30)	11/8	M	22.6 ± 2.9	Recreationally Active	Cycling	1 × (4 × 10 s)	60 s	Mean Power, Peak Power
Zandona et al. (31)	10/10	M	28 ± 5	Trained Cyclists	Cycling	1 × (4 × 30 s)	4 min	Relative Mean Power

M, Male; F, Female; BA: β-Alanine; PLA, Placebo; RSA, Repeated Sprint Ability; LIST, Loughborough Intermittent Shuttle Test; Cross: Crossover design. Rest Interval: Represents the recovery time between sprint repetitions. Values for age are presented as Mean ± SD where available, or approximated based on the group description (e.g., “College-aged”).

### 3.2 Supplementation protocols

The detailed β-alanine supplementation strategies employed across the 17 RCTs are outlined in Table 2. The intervention durations ranged from a minimum of 3 weeks to a maximum of 10 weeks, with the majority of studies adopting a 4–6-week loading phase. Daily dosages varied between 3.2 and 6.4 g/day, resulting in total administered doses ranging from 134.4 to 268.8 g. Notably, nearly half of the trials utilized a patented sustained-release formulation (CarnoSyn™) to mitigate paresthesia, while the remaining studies administered standard gelatin capsules. Placebo substances were appropriately matched and predominantly consisted of dextrose, maltodextrin, or cellulose.

**TABLE 2:** Detailed β-alanine supplementation protocols of the included randomized controlled trials.

Study	Daily dose (g/day)	Duration	Total dose (g)	Supplement form	Placebo substance
Brisola et al. (15)	4.8 (10 days) + 6.4 (18 days)	4 weeks	163.2	Gelatin capsules	Dextrose
Claus et al. (16)	6.4	6 weeks	268.8	Capsules	Dextrose
Cochran et al. (17)	3.2	10 weeks	224	SR Tablets (CarnoSyn™)	Cellulose
Danaher et al. (18)	4.8 (4 weeks) + 6.4 (2 weeks)	6 weeks	224	Capsules	Calcium Carbonate
Ducker et al. (19)	6	4 weeks	168	Capsules	Glucose
Hoffman et al. (20)	6	30 days	180	SR Tablets	Rice Flour
Kresta et al. (21)	∼ 6.0 (0.1 g/kg)	4 weeks	168	Capsules	Dextrose
Milioni et al. (22)	6.4	6 weeks	268.8	Gelatin capsules	Dextrose
Milioni et al. (23)	6.4	6 weeks	268.8	Capsules	Dextrose
Ribeiro et al. (24)	6.4	3 weeks	134.4	SR Tablets (CarnoSyn™)	Maltodextrin
Rosas et al. (25)	4.8	6 weeks	201.6	Capsules	Maltodextrin
Saunders et al. (26)	6.4	4 weeks	179.2	SR Tablets (CarnoSyn™)	Maltodextrin
Saunders et al. (27)	6.4 (4 weeks) + 3.2 (1 week)	5 weeks	201.6	SR Tablets (CarnoSyn™)	Maltodextrin
Smith et al. (28)	6.4	6 weeks	268.8	SR Tablets (CarnoSyn™)	Maltodextrin
Sweeney et al. (29)	4.0 (1 week) + 6.0 (4 weeks)	5 weeks	196	Capsules	Rice Flour
Wang et al. (30)	6.4	4 weeks	179.2	SR Tablets (CarnoSyn™)	Cellulose
Zandona et al. (31)	6.4	4 weeks	179.2	Capsules	Dextrose

SR, Sustained-release formulation (typically designed to reduce paresthesia). Total Dose is calculated as the daily dose multiplied by the number of days of supplementation. CarnoSyn: A patented sustained-release β-alanine formulation used in several studies.

### 3.3 Meta-analysis of repeated sprint ability outcomes

To synthesize the quantitative data while accounting for the varying exercise modes and measurement units, outcomes were categorized into three functional dimensions. The standardized mean differences (SMD) were calculated to determine the overall effect sizes.

- lMean RSA Performance: Analysis of mean performance (e.g., total sprint time, average power) across 17 RCTs revealed that chronic β-alanine supplementation resulted in no significant difference compared to the placebo. The pooled effect size was trivial and statistically non-significant (SMD = −0.018, 95% CI [−0.207 to 0.170], *P* = 0.841). The analysis indicated absolute zero between-study heterogeneity (*I*^2^ = 0%).
- lPeak RSA Performance: For peak anaerobic power or best sprint time (*k* = 10), the meta-analysis demonstrated a small, non-significant effect (SMD = 0.205, 95% CI [−0.073 to 0.483], *P* = 0.130), indicating that maximal power output during initial sprints was not augmented by the intervention (*I*^2^ = 0%).
- lFatigue Index/Decrement: The capacity to attenuate performance decrement across multiple sprints (*k* = 11) was similarly unaffected by β-alanine. The aggregated results showed no significant improvement in fatigue resistance (SMD = −0.020, 95% CI [−0.516–0.476], *P* = 0.929). Notably, moderate between-study heterogeneity was observed for this specific outcome (*I*^2^ = 51.5%), likely reflecting the diverse mathematical formulas utilized to calculate fatigue indices (e.g., decrement scores vs. percentage drop-off) across different protocols.

**FIGURE 2:**
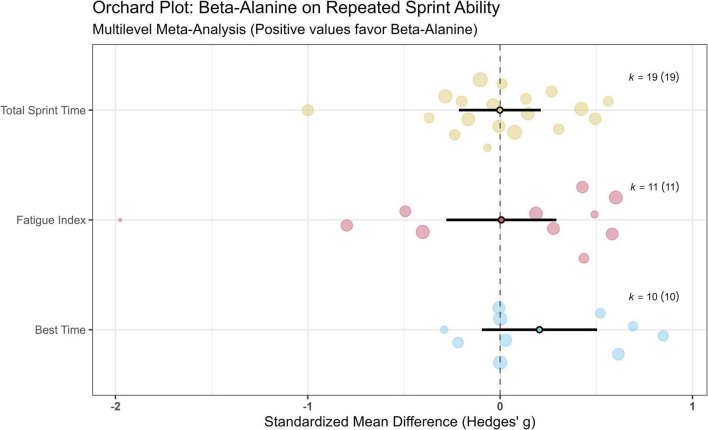
Multilevel meta-analysis evaluating the effects of chronic β-alanine supplementation on the three functional dimensions of Repeated Sprint Ability (RSA). > Data are visualized using Orchard plots for Mean Performance, Peak Performance and Fatigue Decrement. The central point on the main trunk represents the pooled overall effect size [Standardized Mean Difference (SMD), Hedges’ g] derived from the multilevel random-effects model. This central estimate is flanked by thick horizontal bars indicating the 95% confidence intervals (CIs) and thinner extended branches representing the 95% prediction intervals (PIs). Individual effect sizes extracted from the primary studies are depicted as translucent circles (the “orchard”); the area of each circle is scaled proportionally to its analytical precision (inverse variance weight), vividly illustrating the distribution and dependency of multiple outcomes within single cohorts. The vertical dashed line signifies the line of no effect (SMD = 0).

While the multilevel Orchard plots (Figure 2) visualize the synthesized data distribution and prediction intervals, a granular breakdown of the individual effect sizes is provided in Supplementary Figure S1. These traditional forest plots detail the specific standardized mean differences (SMDs), 95% confidence intervals, and relative analytical weights for each primary study, offering a comprehensive view of the underlying data structure across the three functional dimensions.

### 3.4 Certainty of evidence (GRADE) and risk of bias

**FIGURE 3:**
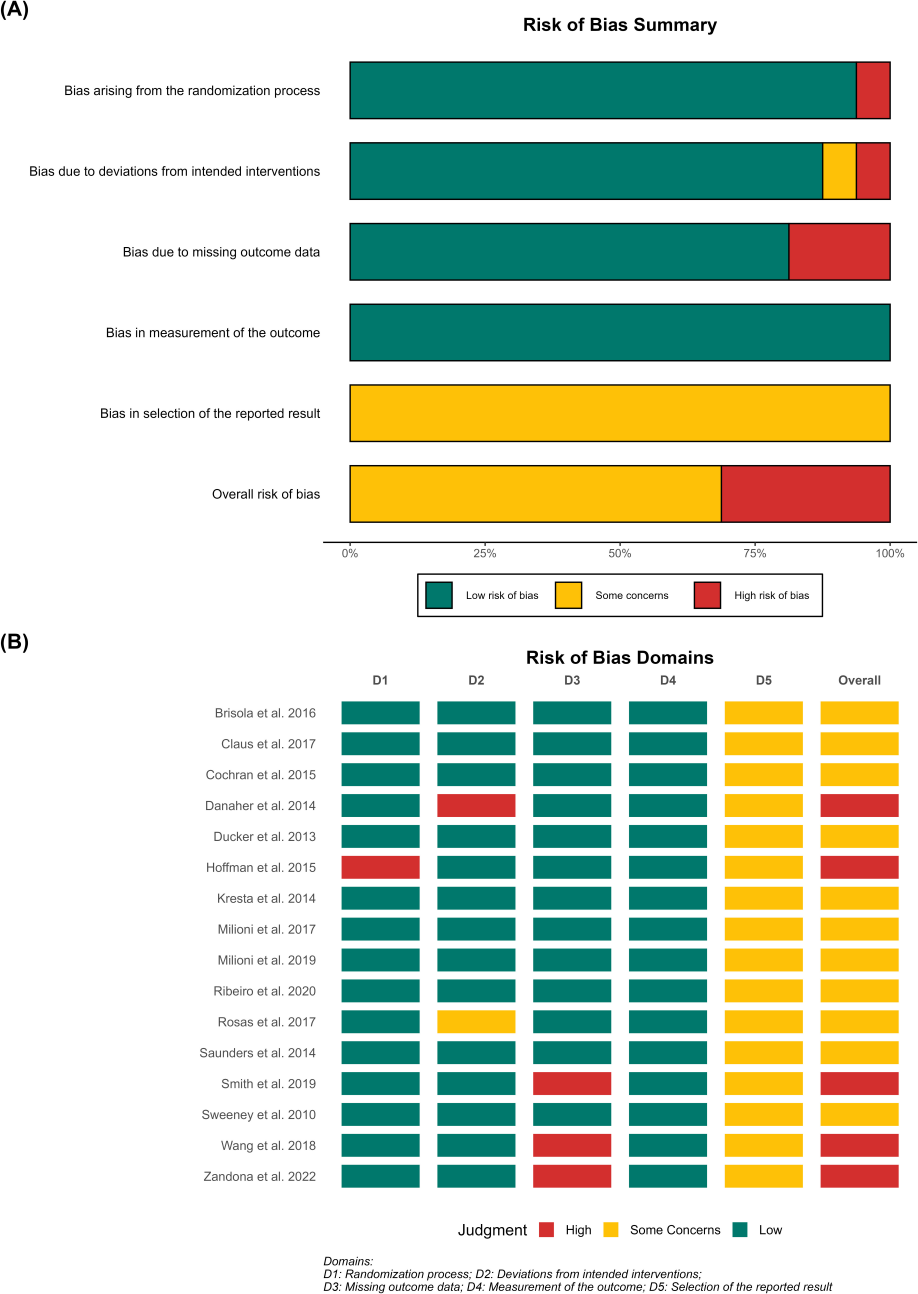
Risk-of-bias assessment of the included randomized controlled trials based on the Cochrane RoB 2.0 tool. **(A)** Risk-of-bias summary showing the percentage of studies rated as low risk, some concerns, or high risk for each domain and overall. **(B)** Study-level traffic-light plot showing domain-specific and overall risk-of-bias judgments for each included trial. D1, randomization process; D2, deviations from intended interventions; D3, missing outcome data; D4, measurement of the outcome; D5, selection of the reported result. Green indicates low risk of bias, yellow indicates some concerns, and red indicates high risk of bias.

The methodological quality of the included RCTs was evaluated using the Cochrane Risk of Bias 2.0 (RoB 2) tool (Figure 3). Overall, no single study was classified as having a completely “Low Risk” of bias. Domain 5 (Selection of the reported result) was the primary contributor to methodological concerns, with all 17 included trials uniformly rated as having “Some Concerns.” This stems primarily from the widespread absence of prospectively registered trial protocols or pre-specified statistical analysis plans, which remains a prevalent limitation within the sports nutrition literature. Conversely, all studies demonstrated a “Low Risk” of bias in Domain 4 (Measurement of the outcome), reflecting the high validity and objective nature of the computerized timing gates and cycle ergometers utilized during RSA testing. Notably, five trials (Danaher et al., Hoffman et al., Smith et al., Wang et al., and Zandona et al.) were ultimately judged to have a “High Risk” of overall bias. These critical downgrades were driven by specific methodological flaws, including inadequate randomization processes (Domain 1), deviations from intended interventions (Domain 2), and substantial missing outcome data without appropriate statistical handling (Domain 3).

Consequently, the overall certainty of the evidence for the effects of β-alanine on RSA outcomes was assessed using the GRADE framework (Table 3). The certainty of evidence for both Mean RSA Performance and Peak RSA Performance was rated as LOW. This was downgraded by one level due to the aforementioned Risk of Bias (driven by the presence of high-risk studies and universal “some concerns” in selective reporting), and further downgraded by one level for Imprecision. The imprecision was characterized by a preponderance of small-study effects (typically *n* < 20 per group), resulting in total sample sizes falling below the optimal information size and yielding wide confidence intervals that cross the line of no effect. Furthermore, the certainty of evidence for the Fatigue Index was downgraded to VERY LOW. In addition to the penalties for risk of bias and severe imprecision (95% CI: −0.52 to 0.48), this specific outcome was further downgraded for Inconsistency. This was justified by the moderate statistical heterogeneity (*I*^2^ = 51.5%) and the conflicting direction of effect sizes among the included trials, likely attributable to the non-equivalent mathematical formulas used to quantify fatigue.

**TABLE 3:** Summary of findings and grade assessment for the certainty of evidence regarding β-alanine supplementation on repeated sprint ability.

Outcomes	Impact	No of participants (studies)	Certainty of the evidence (GRADE)	Relative effect (95% CI)	Comments
Mean RSA performance	β-alanine may result in little to no difference in mean repeated sprint performance. SMD −0.018 (95% CI −0.21 to 0.17)	293(16 RCTs)	⊕⊕○○LOW ^a,b^	SMD: −0.018(trivial effect)	The confidence interval crosses zero and includes both trivial benefit and harm.
Peak RSA performance	β-alanine likely results in little to no difference in peak sprint power. SMD 0.205 (95% CI −0.07 to 0.48)	159(9 RCTs)	⊕⊕○○LOW ^a,b^	SMD: 0.205(trivial effect)	Typically primarily dependent on phosphocreatine (PCr) system, less influenced by pH buffering.
Fatigue index	The effect of β-alanine on fatigue attenuation is uncertain. SMD −0.020 (95% CI −0.52 to 0.48)	182(10 RCTs)	⊕○○○VERY LOW ^a,c,d^	SMD: −0.020(trivial effect)	High heterogeneity observed (*I*^2^ = 52%). Results are inconsistent across studies.

CI, Confidence Interval; SMD, Standardized Mean Difference (Hedges’ g); RCT, Randomized Controlled Trial.

^a^Downgraded one level for Risk of Bias: Most included studies had small sample sizes (*n* < 20 per group), increasing the risk of small-study effects, although blinding was generally adequate.

^b^Downgraded one level for Imprecision: The total sample size is small (< 400 optimal information size), and the 95% CI is wide, crossing the line of no effect and including negligible effects.

^c^Downgraded one level for Inconsistency: Significant heterogeneity was observed (*I*^2^ = 52%), indicating variability in results that cannot be explained by chance alone (likely due to different fatigue calculation formulas used across studies).

^d^Downgraded one level for Imprecision: The 95% CI is very wide (−0.36 to 0.51), indicating a high degree of uncertainty about the magnitude and direction of the effect.

### 3.5 Subgroup, sensitivity, and meta-regression analyses

**FIGURE 4:**
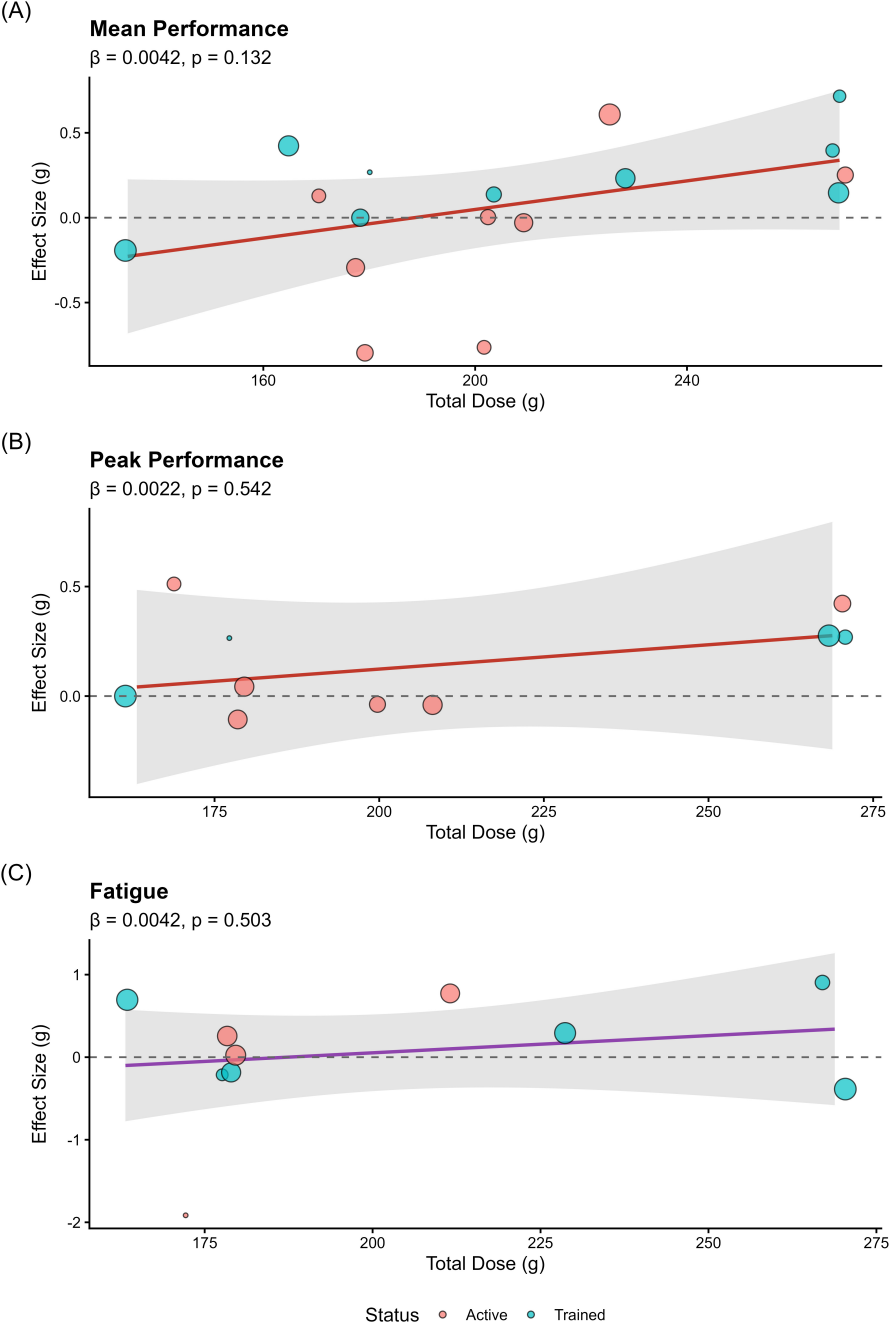
Meta-regression analysis evaluating the dose-response relationship between chronic β-alanine supplementation and repeated sprint ability. Scatter plots depict the relationship between the total administered dose of β-alanine (in grams) and the standardized mean differences (SMD) for **(A)** Mean Performance (*P* = 0.132), **(B)** Peak Performance (*P* = 0.542), and **(C)** Fatigue Decrement (*P* = 0.503). Each circle represents an individual effect size from the included studies, with the size of the circle proportional to the study’s precision (inverse variance weight). The solid line represents the predicted meta-regression fit, surrounded by shaded regions indicating the 95% confidence intervals. The horizontal dashed line denotes the line of no effect (SMD = 0). The flat slopes and non-significant *P*-values across all three functional dimensions confirm the absence of a dose-dependent ergogenic effect.

To explore potential sources of variance and validate the robustness of the null findings, rigorous subgroup and meta-regression analyses were conducted. Meta-regression did not identify a significant dose-response relationship (Figure 4); the total dose of β-alanine (g) did not moderate the effects on mean performance, peak performance, or fatigue attenuation (all *P* > 0.05).

Categorical subgroup analyses further corroborated these findings (Supplementary Table S2). The analyses confirmed that intervention duration (≤ 4 weeks vs. > 4 weeks, P_interaction_ ranging from 0.267 to 0.823), total administered dose (High Dose ≥ 179 g vs. Low Dose < 179 g, P_interaction_ > 0.350), exercise modality (cycling, running, swimming, P_interaction_ > 0.110), and participants’ training status (recreationally active vs. trained athletes, P_interaction_ > 0.210) did not significantly alter the outcomes across any of the functional dimensions. While an isolated within-group significance was observed for fatigue decrement in the swimming subgroup (*P* = 0.023), the overall interaction for exercise modality remained non-significant (P_interaction_ = 0.117), and thus no definitive subgroup effect can be established.

Finally, leave-one-out sensitivity analyses demonstrated that the exclusion of any single study did not shift the pooled SMDs to statistical significance, underscoring the high stability of these null results. Quantitative assessments via Egger’s and Begg’s tests, alongside trim-and-fill analyses, confirmed the absence of publication bias across all functional domains (Supplementary Figure S2).

## 4 Discussion

The present systematic review and multilevel meta-analysis evaluated the efficacy of chronic β-alanine supplementation on repeated sprint ability (RSA). Across 17 randomized controlled trials (RCTs) and three functionally distinct outcome domains (mean performance, peak performance, fatigue decrement), the pooled evidence does not support a meaningful improvement in RSA performance with β-alanine supplementation. Importantly, extensive subgroup and meta-regression analyses suggested that these null effects are not materially moderated by the total administered dose, supplementation duration, or training status—consistent with the broader β-alanine literature showing that ergogenicity is strongly contingent on task duration and the dominant fatigue mechanism, rather than dosing alone once carnosine is meaningfully elevated (8, 10, 32). To conceptualize these findings, a visual summary encompassing the study design, the synthesized null effects across all functional domains, and the proposed bioenergetic mismatch mechanism is provided in the Graphical abstract.

### 4.1 Bioenergetic mismatch: β-alanine versus the ATP–PCR constraint in RSA

The mechanistic rationale for chronic β-alanine supplementation is the elevation of intramuscular carnosine, which contributes to intracellular buffering and can attenuate acid–base perturbations during high glycolytic flux (8). In line with this mechanism, meta-analytic and consensus evidence demonstrates the most reproducible benefits in high-intensity efforts lasting ∼1–4 min, where glycolytic contribution and proton accumulation are substantial (10, 32).

By contrast, RSA protocols are typically composed of very short (e.g., 5–10 s), maximal or near-maximal bouts interspersed with brief recoveries. During the initial sprints, ATP turnover is dominated by rapid phosphocreatine (PCr) hydrolysis, and the immediate constraint on peak power is more tightly linked to PCr availability, excitation–contraction coupling, and metabolite/ionic perturbations such as inorganic phosphate (P_*i*_), rather than to acidosis *per se* (1, 33, 34). Although glycolytic flux and blood lactate typically rise as sprints accumulate, proton accumulation is not necessarily the primary limiter of early peak power output; rather, the relative importance of acid–base disturbance is likely to increase later in the sequence as homeostatic disruption compounds (1, 33). Within this mechanistic framework, it is unsurprising that enhancing buffering capacity via β-alanine yields at most trivial effects on peak performance (SMD = 0.11) in RSA tasks, where the dominant early bottleneck is not H^+^ buffering.

### 4.2 Inter-sprint recovery dynamics: why buffering does not translate to fatigue attenuation

Across many RSA formats, maintaining performance over repeated bouts depends critically on the kinetics of PCr resynthesis and the restoration of contractile function during short recoveries (often 20–60 s) (1, 2). Mechanistic work further indicates that recovery of repeated-sprint performance is strongly coupled to PCr resynthesis, even when muscle pH remains depressed—highlighting that pH normalization is not a prerequisite for meaningful short-term recovery of sprint capacity (11).

While elevated carnosine could theoretically mitigate acidosis-related reductions in force in later repetitions, β-alanine does not directly accelerate oxidative phosphorylation, PCr resynthesis, or the broader set of recovery-limiting perturbations (e.g., P_*i*_-related impairment of cross-bridge function and Ca^2+^ handling) that contribute to power decrement in repeated maximal efforts (33, 34). The present meta-analysis found a fully non-significant effect on fatigue decrement (SMD = 0.07), which aligns with a model in which RSA fatigue is governed primarily by oxidative recovery/PCr restoration and multi-factorial metabolite/ionic stress rather than by intracellular buffering alone (1, 34, 35).

### 4.3 Supplementation protocol and training status: moving beyond “insufficient dose” explanations

Narrative accounts have sometimes attributed inconsistent RSA findings to “suboptimal” β-alanine protocols (e.g., insufficient total dose or duration). However, our meta-regression and subgroup analyses provide limited support for this explanation. Notably, prior meta-analyses indicate that total β-alanine dose (including the often-cited ∼179 g threshold) does not robustly moderate performance outcomes, and that benefits are instead concentrated in tasks where glycolytic acidosis is a prominent constraint (10, 32). Thus, even when carnosine elevation is likely sufficient, the central limitation in RSA may remain mechanistically upstream or orthogonal to buffering—particularly in short-sprint formats where ATP–PCr dynamics and recovery kinetics dominate (1, 11).

We also found no meaningful interaction by training status. Nevertheless, a “ceiling effect” in highly trained cohorts remains biologically plausible: athletes chronically exposed to sprint training often display enhanced repeated-sprint recovery characteristics and metabolite handling, potentially reducing the marginal utility of additional buffering (1, 2). Under such conditions, any β-alanine effect—if present—may be small and difficult to detect without very large, precisely phenotyped samples and tightly standardized RSA tasks. Additionally, while the swimming subgroup demonstrated a statistically significant improvement in fatigue decrement, the overall interaction by exercise modality remained non-significant. Therefore, this isolated finding should be interpreted with caution and warrants further targeted investigation rather than broad generalization.

### 4.4 Methodological considerations and certainty of evidence

A key strength of this review is the multilevel meta-analytic framework, which explicitly addresses dependence among multiple effect sizes derived from the same study (e.g., peak power, mean power, and fatigue metrics from one cohort). This approach is aligned with contemporary guidance showing that ignoring within-study dependence can underestimate standard errors and inflate the apparent precision of pooled effects (14, 36). The finding of minimal residual heterogeneity for the primary effects (*I*^2^ ≈ 0%) should therefore be interpreted as reflecting both consistent null effects and appropriate modeling of dependency rather than as evidence that the underlying RSA literature is methodologically homogeneous.

In contrast, certainty for fatigue outcomes was downgraded (GRADE “Very Low”) due to the moderate statistical heterogeneity observed for this specific outcome (*I*^2^ = 51.5%). This inconsistency arises primarily because fatigue indices are calculated using non-equivalent formulas (e.g., percent decrement vs. fatigue index) (37) and because sprint/rest structures vary substantially across trials—both of which can materially influence fatigue expression (38, 39). For instance, percentage drop-off algorithms often yield different variance profiles compared to absolute decrement scores, which can artificially inflate between-study heterogeneity. To resolve this dispersion, we explicitly recommend that future trials adopt standardized fatigue calculation formulas and transparent reporting practices to improve cross-study comparability.

Following GRADE guidance, additional downgrades were warranted for risk of bias and imprecision, as many RCTs were small (often *n* < 20 per group) with wide confidence intervals (40, 41). Regarding the universal “Some Concerns” in Domain 5 due to a lack of prospective registration, it must be acknowledged that this limits the overall certainty of evidence as it leaves room for selective reporting. However, it is also highly pertinent that the inherent objectivity of RSA outcomes—which are almost exclusively measured via mechanized timing gates or computerized cycle ergometers—partially mitigates the risk of *post hoc* data manipulation compared to more subjective performance metrics. Future research should prioritize adequately powered, pre-registered trials with standardized RSA protocols, harmonized fatigue calculations, and transparent reporting of sprint and recovery parameters.

Furthermore, it is important to acknowledge that the majority of the included studies recruited male participants, with only three trials exclusively involving females. This gender imbalance represents a limitation in the current literature, and future research should aim for greater inclusion of female athletes to confirm whether these null effects are universally applicable across sexes.

### 4.5 Practical applications

Collectively, the current evidence indicates that chronic β-alanine supplementation is unlikely to confer a meaningful ergogenic advantage for RSA, particularly when performance is constrained primarily by ATP–PCr availability and inter-sprint recovery kinetics rather than by intracellular buffering. Practically, when sporting demands are dominated by short-duration repeated sprinting (e.g., discrete phases of team and racket sports), β-alanine should not be prioritized as a primary supplement strategy. Instead, resources may be better directed toward interventions with stronger mechanistic alignment to RSA constraints. For example, creatine monohydrate offers superior bioenergetic compatibility with RSA; by significantly elevating intramuscular phosphocreatine (PCr) stores, it directly bolsters the primary energy substrate required for initial maximal efforts. More importantly, it accelerates PCr resynthesis via the creatine kinase reaction during the characteristically brief recovery intervals, thereby maintaining rapid ATP turnover and delaying power decrement across consecutive sprints (42, 43). Similarly, acute caffeine supplementation directly targets the neuromuscular constraints of repeated high-intensity actions. Through its antagonism of adenosine receptors, caffeine centrally enhances motor unit recruitment, sustains central nervous system (CNS) drive, and potentially improves intracellular calcium (Ca^2+^) handling, thereby counteracting the integrated neural and peripheral fatigue that typically limits RSA (44–46).

### 4.6 Future directions

Consequently, we propose that future RSA research should pivot away from interventions focused strictly on intracellular buffering. Instead, sports nutrition and physiological investigations should prioritize strategies designed to accelerate metabolic clearance (e.g., inorganic phosphate removal) and enhance muscle reoxygenation and oxidative recovery kinetics during the characteristically brief rest intervals of repeated sprint protocols. Furthermore, given the vast inter-individual variability in repeated-sprint fatigue, future studies should move beyond generalized group-level analyses. Identifying distinct metabolic phenotypes among athletes and utilizing advanced predictive modeling to understand how specific physiological traits dictate recovery profiles will be crucial for developing highly personalized ergogenic and training recommendations. Methodologically, it remains imperative that subsequent trials adopt standardized, prospectively registered RSA protocols with harmonized fatigue calculations and transparent reporting of both sprint and recovery parameters to facilitate more robust quantitative syntheses in the future.

## 5 Conclusion

In conclusion, the available evidence suggests that chronic β-alanine supplementation is unlikely to provide a meaningful ergogenic benefit for repeated sprint ability (RSA), although this interpretation should be viewed in light of the small sample sizes and methodological limitations of the current literature. The pooled estimates showed trivial, non-significant effects across all functional dimensions—mean work capacity, maximal peak power, and fatigue attenuation—irrespective of the total administered dose, supplementation duration, exercise modality, or the athletes’ training status. While β-alanine is a proven intracellular buffer via muscle carnosine synthesis, this mechanism may be less well aligned with the dominant metabolic constraints of short-duration repeated sprinting. The capacity to sustain maximal power output across consecutive brief sprints is predominantly dictated by phosphocreatine (PCr) depletion and inter-sprint oxidative recovery kinetics, rather than severe glycolytic acidosis. Consequently, sports nutrition practitioners, dietitians, and coaches may wish to reconsider the prioritization of β-alanine when competition profiles or conditioning objectives are strictly defined by intermittent, short-duration maximal efforts. To effectively optimize RSA, future nutritional strategies may be better directed toward interventions that facilitate rapid PCr resynthesis and support metabolic recovery during brief rest intervals.

## Data Availability

The original contributions presented in the study are included in the article/Supplementary material, further inquiries can be directed to the corresponding authors.
