# Supplementary material for: No ergogeniceffect of β-alanine on repeated sprint ability: a systematic review and multilevel meta-analysis of randomized controlled trials
Source: Front Nutr. 2026 Mar 26;13:1818755. doi: 10.3389/fnut.2026.1818755 (PMC13061858; doi:10.3389/fnut.2026.1818755)
Supplement: Supplementary file 1 [file Supplementary_file_1.docx]

# Supplemental Online Content

Table S1. Search Strategy.

Table S2. Subgroup analyses of the effects of chronic β-alanine supplementation on the functional dimensions of Repeated Sprint Ability.

Figure S1. Traditional forest plots detailing the individual and pooled effect sizes of chronic β-alanine supplementation on RSA outcomes.

Figure S2. Funnel plots for the assessment of publication bias.

# Table S1. Search Strategy.

| #1  Pubmed  404 Results | ("beta-Alanine"[Mesh] OR "beta-alanine"[Title/Abstract] OR "beta alanine"[Title/Abstract] OR "β-alanine"[Title/Abstract] OR "3-aminopropionic acid"[Title/Abstract] OR "carnosine"[Title/Abstract]) AND ("Athletic Performance"[Mesh] OR "High-Intensity Interval Training"[Mesh] OR "sprint*"[Title/Abstract] OR "repeated sprint*"[Title/Abstract] OR "RSA"[Title/Abstract] OR "sprint interval*"[Title/Abstract] OR "SIT"[Title/Abstract] OR "intermittent exercise"[Title/Abstract] OR "high intensity"[Title/Abstract] OR "anaerobic"[Title/Abstract] OR "Wingate"[Title/Abstract] OR "ergogenic"[Title/Abstract]) |
| --- | --- |
| #2  Web of Science | TS=("beta-alanine" OR "beta alanine" OR "β-alanine" OR "3-aminopropionic acid" OR "carnosine")  AND  TS=("sprint*" OR "repeated sprint*" OR "RSA" OR "sprint interval*" OR "SIT" OR "high-intensity interval" OR "HIIT" OR "intermittent exercise" OR "anaerobic power" OR "Wingate") |
| #3  SPORTDiscus | (TI ("beta-alanine" OR "beta alanine" OR "β-alanine" OR "carnosine") OR AB ("beta-alanine" OR "beta alanine" OR "β-alanine" OR "carnosine"))  AND  (TI ("sprint*" OR "repeated sprint*" OR "RSA" OR "interval training" OR "SIT" OR "HIIT" OR "intermittent" OR "anaerobic") OR AB ("sprint*" OR "repeated sprint*" OR "RSA" OR "interval training" OR "SIT" OR "HIIT" OR "intermittent" OR "anaerobic")) |
| #4  Cochrane Library  85 | #1 ("beta-alanine" OR "beta alanine" OR "carnosine"):ti,ab,kw  #2 ("sprint*" OR "repeated sprint*" OR "interval training" OR "high intensity" OR "intermittent exercise"):ti,ab,kw  #3 #1 AND #2 |
| Embase | 1. exp beta alanine/ or exp carnosine/  2. (beta-alanine or "beta alanine" or β-alanine or carnosine).ti,ab.  3. 1 or 2  4. exp sprint/ or exp high intensity interval training/ or exp anaerobic capacity/  5. (sprint* or "repeated sprint*" or RSA or "sprint interval*" or SIT or HIIT or "intermittent exercise").ti,ab.  6. 4 or 5  7. 3 and 6 |
| Scopus | TITLE-ABS-KEY (  ( "beta-alanine" OR "beta alanine" OR "carnosine" )  AND  ( "sprint*" OR "repeated sprint*" OR "RSA" OR "sprint interval*" OR "SIT" OR "high-intensity interval" OR "intermittent exercise" OR "anaerobic" ) ) |

# Table S2. Subgroup analyses of the effects of chronic β-alanine supplementation on the functional dimensions of Repeated Sprint Ability.

| **Outcome / Subgroup Variable** | **Group Level** | **k** | **Pooled SMD (95% CI)** | **P-value (Within)** | **I2 (%)** | **Pinteraction​ (Between)** |
| --- | --- | --- | --- | --- | --- | --- |
| **Mean Performance** |  |  |  |  |  |  |
| Total Dose | High Dose (≥ 179g) | 14 | 0.07 (-0.18, 0.32) | 0.602 | 0.00% | 0.892 |
|  | Low Dose (< 179g) | 3 | 0.11 (-0.40, 0.61) | 0.68 | 0.00% |  |
| Duration | ≤ 4 Weeks | 7 | -0.08 (-0.43, 0.27) | 0.669 | 0.00% | 0.267 |
|  | > 4 Weeks | 10 | 0.18 (-0.11, 0.48) | 0.225 | 0.00% |  |
| Exercise Modality | Cycling | 7 | -0.09 (-0.49, 0.30) | 0.641 | 22.00% | 0.38 |
|  | Running | 8 | 0.13 (-0.20, 0.47) | 0.437 | 0.00% |  |
|  | Swimming | 2 | 0.41 (-0.24, 1.06) | 0.215 | 0.00% |  |
| Training Status | Trained | 9 | 0.21 (-0.10, 0.52) | 0.186 | 0.00% | 0.215 |
|  | Active | 8 | -0.08 (-0.43, 0.27) | 0.645 | 11.40% |  |
| **Peak Performance** |  |  |  |  |  |  |
| Total Dose | High Dose (≥ 179g) | 8 | 0.13 (-0.21, 0.46) | 0.453 | 0.00% | 0.841 |
|  | Low Dose (< 179g) | 2 | 0.20 (-0.45, 0.85) | 0.54 | 0.00% |  |
| Duration | ≤ 4 Weeks | 5 | 0.11 (-0.31, 0.53) | 0.612 | 0.00% | 0.823 |
|  | > 4 Weeks | 5 | 0.18 (-0.24, 0.60) | 0.406 | 0.00% |  |
| Exercise Modality | Cycling | 4 | 0.08 (-0.39, 0.54) | 0.745 | 0.00% | 0.893 |
|  | Running | 4 | 0.23 (-0.25, 0.72) | 0.34 | 0.00% |  |
|  | Swimming | 2 | 0.11 (-0.54, 0.75) | 0.743 | 0.00% |  |
| Training Status | Trained | 4 | 0.19 (-0.28, 0.65) | 0.435 | 0.00% | 0.817 |
|  | Active | 6 | 0.11 (-0.27, 0.50) | 0.559 | 0.00% |  |
| **Fatigue Index** |  |  |  |  |  |  |
| Total Dose | High Dose (≥ 179g) | 9 | 0.14 (-0.18, 0.45) | 0.395 | 0.00% | 0.357 |
|  | Low Dose (< 179g) | 2 | -0.57 (-3.13, 1.99) | 0.661 | 91.40% |  |
| Duration | ≤ 4 Weeks | 6 | -0.15 (-0.79, 0.49) | 0.648 | 61.30% | 0.333 |
|  | > 4 Weeks | 5 | 0.26 (-0.22, 0.74) | 0.293 | 25.00% |  |
| Exercise Modality | Cycling | 5 | -0.05 (-0.87, 0.77) | 0.903 | 72.70% | 0.117 |
|  | Running | 4 | -0.23 (-0.71, 0.24) | 0.341 | 0.00% |  |
|  | Swimming | 2 | 0.78 (0.11, 1.45) | 0.023* | 0.00% |  |
| Training Status | Trained | 6 | 0.18 (-0.24, 0.60) | 0.409 | 17.10% | 0.512 |
|  | Active | 5 | -0.13 (-0.94, 0.68) | 0.751 | 71.10% |  |

k, number of effect sizes included in the subgroup; SMD, Standardized Mean Difference (Hedges' g); CI, Confidence Interval; I², statistic describing the percentage of variation across studies that is due to true heterogeneity rather than chance; P-value (Within), statistical significance of the pooled effect size within the specific subgroup; P_interaction (Between), statistical significance for the test of subgroup differences (moderator effect). Total Dose was dichotomized based on the median value (179g) across the included primary studies. * Indicates statistical significance within the specific subgroup (P < 0.05); however, the overall between-group interaction for exercise modality remained non-significant (P_interaction_ = 0.117).


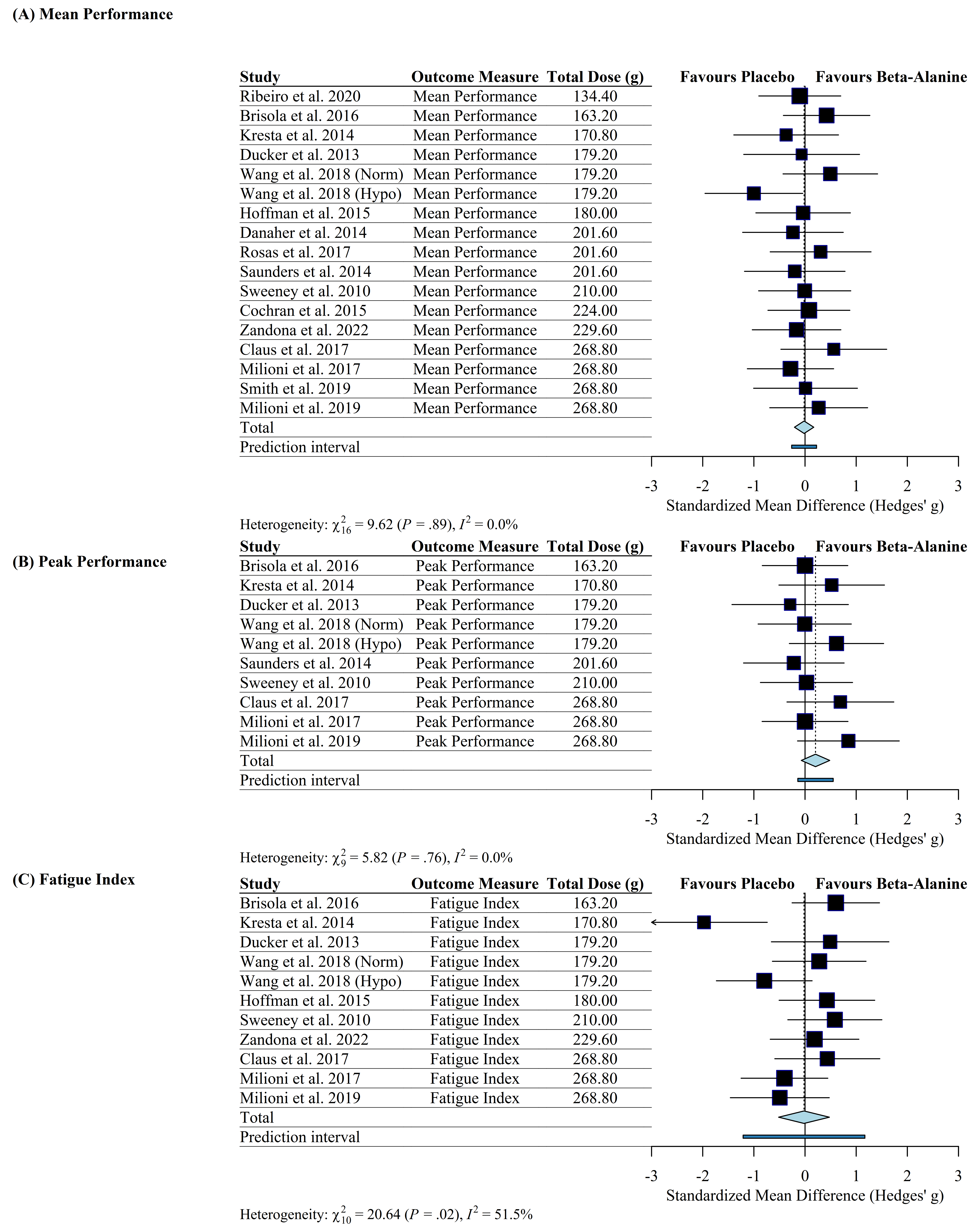


# Figure S1. Traditional forest plots detailing the individual and pooled effect sizes of chronic β-alanine supplementation on RSA outcomes. The plots display the standardized mean differences (SMD, Hedges' g) and 95% confidence intervals (CIs) for each included trial across the three functional dimensions (Mean Performance, Peak Performance, and Fatigue Decrement). Black squares represent the effect size of individual studies, with the area proportional to the analytical weight. The black diamonds indicate the overall pooled effect sizes based on the multilevel model.


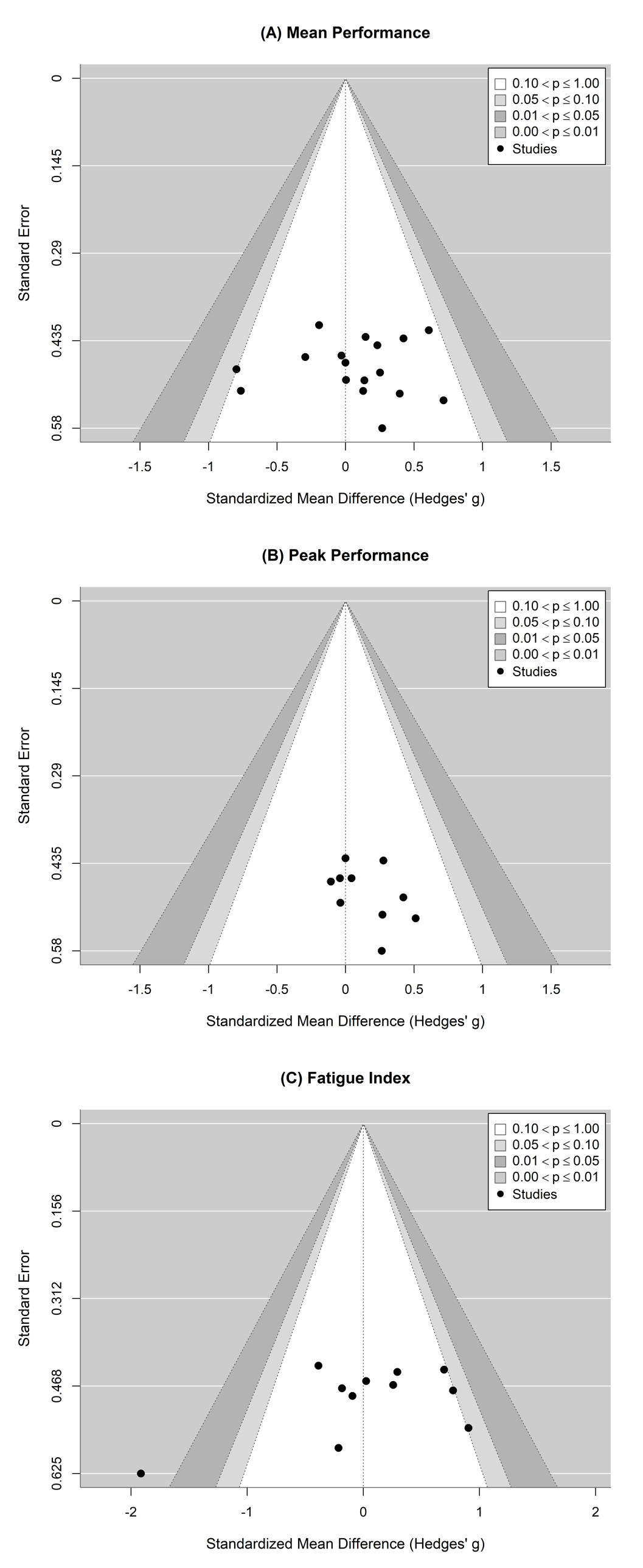


# Figure S2. Funnel plots for the assessment of publication bias. Visual inspection of the funnel plots for (A) Mean Performance、 (B) Peak Performance and (C) Fatigue Index, complemented by Duval and Tweedie’s trim-and-fill analysis, revealed symmetry around the pooled effect estimates, indicating the absence of small-study effects or publication bias (Missing k = 0).
